# Associations of the Intake of Individual and Multiple Flavonoids with Metabolic Dysfunction Associated Steatotic Liver Disease in the United States

**DOI:** 10.3390/nu17020205

**Published:** 2025-01-07

**Authors:** Chen Wang, Mengchu Li, Jiali Zhang, Hongguang Li, Yue Li, Siyu Huang, Huilian Zhu, Zhaoyan Liu

**Affiliations:** 1 Department of Nutrition, School of Public Health, Sun Yat-sen University, 74 Zhong Shan Road 2, Guangzhou 510080, China; wangch365@mail2.sysu.edu.cn (C.W.); limch55@mail2.sysu.edu.cn (M.L.); zhangjli6@mail2.sysu.edu.cn (J.Z.); huangsy9@mail2.sysu.edu.cn (S.H.); zhuhl@mail.sysu.edu.cn (H.Z.); 2 Guangdong Provincial Key Laboratory of Food, Nutrition and Health, School of Public Health, Sun Yat-sen University, Guangzhou 510080, China; 3 Department of Clinical Nutrition, Zhongshan City People’s Hospital, Zhongshan 528400, China; zslihg2293@163.com (H.L.); 15377842591@163.com (Y.L.)

**Keywords:** flavonoids, MASLD, NHANES, WQS, NAFLD

## Abstract

Background: Evidence regarding the individual and combined impact of dietary flavonoids on the risk of metabolic dysfunction associated with steatotic liver disease (MASLD) remains scarce. Our objective is to evaluate the association between individual and multiple dietary flavonoids with MASLD in adults. Methods: Data sets were obtained from the National Health and Nutrition Examination Survey (NHANES), 2017–2018. In total, 2581 participants aged over 18 years, with complete information on dietary flavonoid intake, MASLD, and covariates, were included. Flavonoid intake was energy-adjusted using the residual method. Logistic regression analysis was employed to examine the impact of total flavonoid intake on MASLD. Weighted quantile sum (WQS) analyses were used to evaluate the combined and individual effects of flavonoids on MASLD and to identify the predominant types with the most significant contribution to MASLD prevention. Results: The highest tertile of total flavonoid intake was associated with a 29% reduction in the risk of MASLD compared to the lowest tertile after multivariable adjustments (OR: 0.71, 95% CI: 0.51–0.97). The WQS analysis revealed that anthocyanidins, flavones, and flavanones were the most critical contributors among six subclasses (weights = 0.317, 0.279, and 0.227, respectively) and naringenin, apigenin, and delphinidin were the most critical contributors among 29 monomers. (weights = 0.240, 0.231, and 0.114, respectively). Also, a higher intake of anthocyanidins, flavones, naringenin, apigenin, and delphinidin was linked to a reduced risk of MASLD (*p* < 0.05).Conclusions: Our findings suggested that a higher flavonoid intake is associated with a lower risk of MASLD, with anthocyanidins, flavones, flavanones, naringenin, apigenin, delphinidin, and myricetin contributing most to the protective effects of flavonoids.

## 1. Introduction

Recently, due to the stigmatized term “fatty”, international experts have proposed a new terminology, metabolic dysfunction associated steatotic liver disease (MASLD), to replace nonalcoholic fatty liver disease (NAFLD) [1]. The diagnosis of MASLD is based on the presence of steatotic liver disease combined with at least one of the five cardiometabolic risk factors (obesity, glycaemic dysregulation, high blood pressure, high triglycerides, and low high-density lipoprotein [HDL] cholesterol), which can more accurately identify individuals at high risk for potential metabolic diseases [1,2]. Compared to the general population, patients with MASLD exhibit elevated rates of disease-specific and overall mortality [3]. Globally, the prevalence of MASLD reached 32.4% in 2022, and it is also the most common cause of chronic liver disease in the United States [4,5]. Without intervention, the prevalence of MASLD is projected to increase to 55.7% by 2040, leading to a huge burden on society [6]. In addition, no specific drug has been approved for the therapy of MASLD up to now. Exploring preventive strategies targeting MASLD risk factors is imminently needed to reduce its prevalence, among which cost-effective modifiable dietary factors are of particular interest [7,8].

Pathologically, MASLD is a complex and multifactorial disease characterized primarily by excess lipid accumulation, followed by hepatocyte toxicity, oxidative stress, insulin resistance, and inflammation [9,10,11]. Flavonoids are natural polyphenols that can be found in teas, vegetables, and fruits. Generally, based on their structures, flavonoids can be classified into six subclasses, including anthocyanins, isoflavones, flavones, flavanones, flavan-3-ols, and flavonols [12]. Due to their beneficial effects on glucose and lipid metabolism, inflammation, and oxidative stress, their effects on liver steatosis have attracted much attention [13,14]. For example, a recent prospective cohort study revealed that a high flavonoid intake was associated with a lower risk of NAFLD in elderly, overweight, and obese populations [15]. Additionally, experimental studies demonstrated that several types of flavonoids, including daidzein [16], cyanidin [17], epigallocatechin [18], eriodictyol [19], apigenin [20], and quercetin [21], exhibited satisfactory effects in ameliorating liver steatosis.

Although compelling evidence has illustrated the protective effects of flavonoids against liver steatosis, no study has investigated the association of dietary flavonoid intake with the risk of MASLD. More importantly, several studies have reported inconsistent results. For example, there was no significant association between total flavonoid intake and NAFLD risk in a cross-sectional study [22], and results from randomized clinical trials remained controversial [23]. These inconsistent conclusions were largely due to that most of the previous studies focused on one single monomer or subclass of flavonoids.

However, in the real world, we typically consume various flavonoids simultaneously, which could lead to interactions among different flavonoids. Traditional statistical methods may limit the exploration of the mixed effects of different flavonoids. Given the pattern of simultaneous intake, the high correlation among flavonoids, and their complex interactions, a specialized approach is necessary to assess the mixed effects. Therefore, our study focuses on the mixed effects of multiple flavonoid subclasses on MASLD and aims to identify which specific flavonoids have the most significant impact. Additionally, the inconsistent conclusions may also stem from the different detection methods for steatotic liver disease. Generally, liver biopsy is considered the gold standard for assessing NAFLD. However, it is not suitable for large-scale population screening due to its invasive nature. Alternative detection methods, including the hepatic steatosis index, fatty liver index, and NAFLD liver fat score, which are based on individual conditions and blood biomarkers, have several drawbacks such as limited sensitivity, specificity, and accessibility. Transient elastography can measure the ultrasound attenuation related to the presence of hepatic steatosis by using a vibrating tip contacting the skin, which is an effective approach to evaluate fatness in the liver. In contrast to liver biopsy, transient elastography offers a non-invasive, convenient, and rapid way to evaluate steatotic liver disease, yet it has been applied to diagnosis in large populations in only a few studies.

Therefore, in this study, we aimed to integrate a traditional method (logistic regression analysis) and an advanced machine learning technique (weighted quantile sum [WQS]), to evaluate the associations between the intake of individual and mixed flavonoids and MASLD, which was determined by liver ultrasound transient elastography among a nationwide population in the United States.

## 2. Methods

### 2.1. Study Population

**Figure 1:**
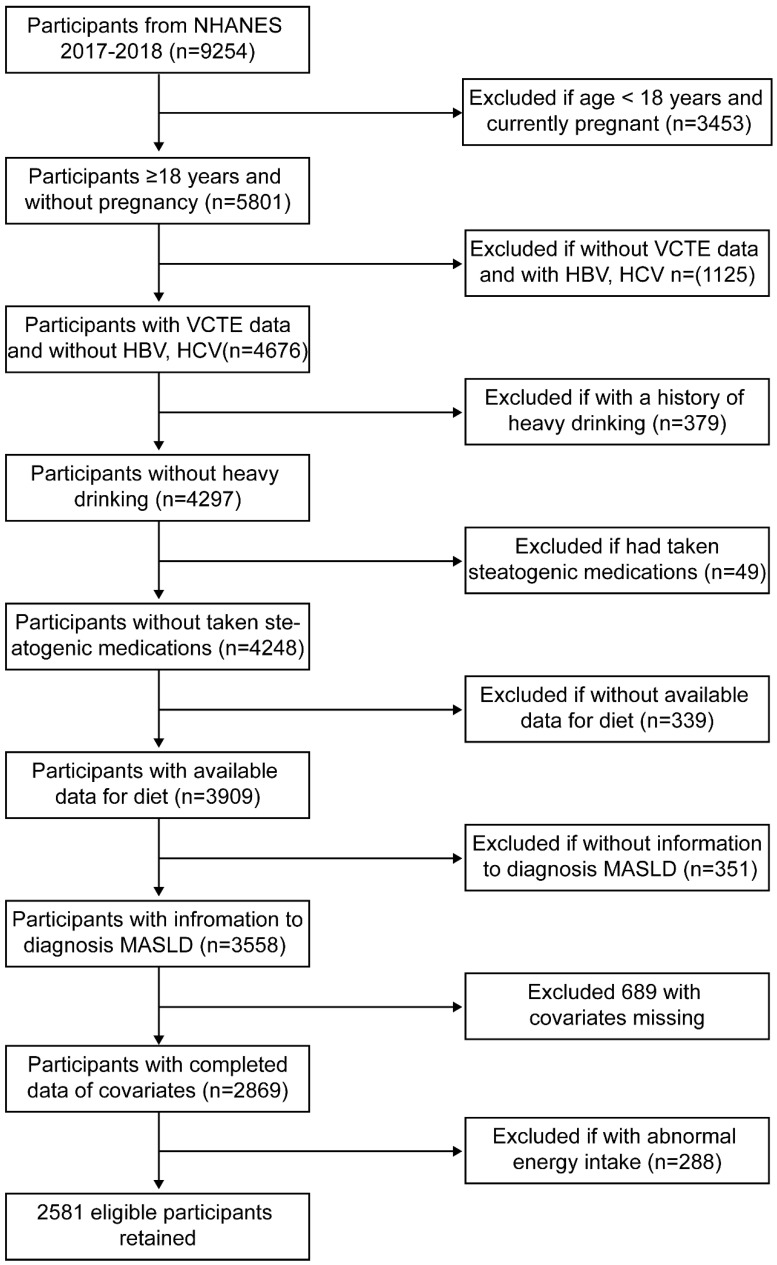
Flow chart of participants included in the final analysis (N = 2581). Abbreviations: NHANES, National Health and Nutrition Examination Survey; VCTE, vibration-controlled transient elastography; HBV, hepatitis B virus; HCV, hepatitis C virus; MASLD, metabolic dysfunction associated steatotic liver disease.

The data of all participants used in this study were obtained from the National Health and Nutrition Examination Survey (NHANES) and the United States Department of Agriculture’s Food and Nutrient Database for Dietary Studies (FNDDS), which can be accessed at https://www.cdc.gov/nchs/nhanes/ (accessed on 9 December 2024) and https://www.ars.usda.gov/ (accessed on 9 December 2024), respectively. We included data from the 2017 to 2018 cycle, with a total of 9254 participants. Subsequently, we excluded individuals with any of the following conditions: (1) age < 18 years; (2) currently pregnant; (3) without available diet data; (4) without available vibration-controlled transient elastography data; (5) had a history of hepatitis B or C virus infection; (6) had a history of heavy drinking (≥14 drinks/week for men or ≥7 drinks/week for women); (7) had taken steatogenic medications; (8) diagnosed with other steatotic liver diseases (SLD), including metabolic dysfunction–associated steatotic liver disease (MetALD), or other combination aetiology, cryptogenic SLD and other specific aetiology SLD; (9) missing data on selected covariates; and (10) extreme energy intake (highest and lowest 5% of energy intake). Finally, a total of 2581 participants remained in our study for further investigation (Figure 1).

### 2.2. Definition of MASLD

The efficacy of elastography in evaluating liver steatosis has been established. We utilized the FibroScan model 502 V2 Touch, equipped with either a medium or extra-large probe, to measure the controlled attenuation parameter (CAP), which serves as an objective indicator of hepatic steatosis. Skilled technicians conducted the examinations at the NHANES Mobile Examination Center (MEC).

In our study, a CAP score equal to or greater than 302 dB/m was considered a steatotic liver diseases [24]. Patients with steatotic liver who met any of the following five criteria and had no other causes of steatosis were diagnosed as MASLD [1]: (1) body mass index (BMI) ≥ 25 kg/m^2^ or waist circumference (WC) > 94 cm for males and >80 cm for females; (2) fasting serum glucose ≥ 5.6 mmol/L (100 mg/dL) or haemoglobin A1C ≥ 5.7% (39 mmol/L) or type 2 diabetes or treatment for type 2 diabetes; (3) blood pressure > 130/85 mmHg or specific antihypertensive drug treatment; (4) plasma triglycerides > 1.70 mmol/L (150 mg/dL) or lipid-lowering treatment; and (5) plasma HDL-cholesterol ≤ 1.0 mmol/L (40 mg/dL) for males and ≤1.3 mmol/L (50 mg/dL) for females or lipid-lowering treatment. Other patients with steatotic liver were diagnosed with MetALD, or other combination aetiology, cryptogenic SLD, and other specific aetiology SLD.

### 2.3. Assessment of Dietary Flavonoid Intake

Trained interviewers utilized a validated 24 h food recall method to assess dietary intake at the MEC, and then conducted a second dietary recall via telephone 3–10 days later. The average dietary intakes from the two distinct days were calculated to represent their dietary status. For participants lacking dietary data from the second recall, only the values from the initial recall were utilized. Briefly, we obtained dietary data for two distinct days from 2577 participants and for a single day from 274 participants. According to the quality and completeness of a survey participant’s response to the dietary recall section, only reliable data assessed by investigators were included in our analysis. FNDDS was used to calculate dietary flavonoid intakes. Flavonoids encompass six subclasses, namely isoflavones, anthocyanins, flavan-3-ols, flavanones, flavones, and flavonols. Isoflavones consist of daidzein, genistein, and glycitein. Anthocyanins consist of cyanidin, delphinidin, malvidin, pelargonidin, peonidin, and petunidin. Flavan-3-ols consist of (−)-epicatechin, (−)-epicatechin 3-gallate, (−)-epigallocatechin, (−)-epigallocatechin 3-gallate, (+)-catechin, (+)-gallocatechin, theaflavin, theaflavin-3,3′-digallate, theaflavin-3′-gallate, theaflavin-3-gallate, and thearubigins. Flavanones consist of eriodictyol, hesperetin, and naringenin. Flavones consist of apigenin and luteolin. Flavonols consist of isorhamnetin, kaempferol, myricetin, and quercetin.

### 2.4. Covariates

The researchers conducted face-to-face interviews to gather demographic information, including age, sex (males and females), ethnicity (non-Hispanic white, non-Hispanic black, Mexican American, and others), and education level (less than high school, high school or equivalent, and college or above). The family income-to-poverty ratio was calculated by dividing family (or individual) income by the poverty guidelines specific to the survey year. Information focused on lifestyle was also collected. Alcohol drinking status and smoking status were both divided into three categories. Non-drinkers were defined as participants who reported consuming less than 12 alcoholic drinks each year, low to moderate drinkers were defined as consuming less than 14 drinks/week for men, or less than 7 drinks/week for women. Never smokers were defined as participants who had smoked less than 100 cigarettes in their entire lifetime. Former smokers were participants who had ceased smoking before the interview, while current smokers were those who had smoked over 100 cigarettes in their entire lifetime and were still smoking at the time of the interview. The Healthy Eating Index 2015 (HEI-2015) was utilized to assess the degree of adherence to the Dietary Guidelines for Americans [25]. The HEI-2015 score ranged from 0 to 100, with a higher score reflecting better diet quality. Participants were considered to have regular physical activity if they reported engaging in any vigorous, moderate, or muscle-strengthening activities for at least 10 min in the past 30 days. Medical conditions were also considered. Blood collection and body and blood pressure measurements were conducted in the MEC. All the examination and laboratory procedure manuals have been described in detail [26,27]. Each of the following criteria was a value of 1, and the sum of the scores was defined as the metabolic syndrome score: (1) WC ≥ 88 cm for women or ≥102 cm for men; (2) triglyceride ≥ 150 mg/dL or taking lipid-modifying medication; (3) HDL-Cholesterol < 40 mg/dL for men or <50 mg/dL for women or taking lipid-modifying medication; (4) systolic blood pressure ≥ 130 mmHg or diastolic blood pressure ≥ 85 mmHg or taking anti-hypertensive drugs; and (5) fasting blood glucose (FBG) ≥ 100 mg/dL or taking antidiabetic drug (including insulin). Sleep disorders and insufficient nighttime sleep duration (<6 h) were the primary issues we considered when assessing sleep quality. Low, moderate, and high sleep quality were defined as having two, one, or none of the above conditions, respectively. Cardiovascular diseases (CVDs) refer to self-reported diagnoses of stroke, angina, heart attack, coronary heart disease, or congestive heart failure in the study.

### 2.5. Statistical Analysis

Given the complex, multi-stage, cluster-sampling design utilized by NHANES, we applied appropriate sample weight to correct the data. Daily flavonoid intakes for each participant were adjusted for total energy intake using the residual method [28]. The residual method for energy adjustment involved calculating the difference between the observed and predicted energy intake using a regression model. The residual was then applied to adjust flavonoid values, ensuring an accurate representation of dietary flavonoid intake. The energy-adjusted flavonoid intake was utilized in all the following analyses. Participants were divided into three groups based on tertiles of energy-adjusted dietary intake of total flavonoids. In the descriptive analysis, continuous variables and categorical variables were described as means and standard deviation (SD), counts and frequencies, respectively. One-way ANOVA and Chi-squared tests were used to compare the differences in basic characteristics as appropriate. A Pearson correlation analysis was used to estimate the bivariate associations between flavonoids.

A logistic regression analysis was conducted to investigate the effect of total flavonoid intake on the risk of MASLD. The minimally adjusted model (model 1) was adjusted for age and sex. The fully adjusted model (model 2) was adjusted for age, sex, ethnicity, smoking status, alcohol drinking status, education level, family poverty impact ratio, metabolic syndrome score, prevalence of CVD, sleep quality, HEI-2015 score, and regular physical activity.

WQS regression analyses were utilized to assess the combined and individual impacts of flavonoid mixtures on MASLD by assigning corresponding weights. We applied bootstrapping with 1000 iterations to construct WQS indexes in both positive and negative directions. The WQS indexes represent the mixed effects of flavonoid intake on MASLD. When the WQS indexes were significant, the corresponding weights were assessed to identify the relative contributions of each flavonoid subclass within the WQS index to MASLD. All flavonoids were transformed into tertiles, and the dataset was randomly divided, with 40% assigned to the training set and the remaining 60% serving as the validation set. The weights allocated to each flavonoid reflected its contribution to MASLD.

Based on the results of the WQS analyses, flavonoids with weights exceeding the selection threshold parameter of 1/6 or 1/29 (corresponding weight > 1/number of mixtures) were considered as a significant contribution to MASLD. Participants were classified into three groups based on the tertiles of each flavonoid intake. The logistic regression analyses were conducted to evaluate the effects of predominant flavonoids on MASLD and its diagnostic criteria, after additionally adjusting for the intake of the higher-level flavonoid class.

Additional analyses stratified by the above-mentioned confounding factors were conducted to examine the modification effect. Interactions were thereafter evaluated using the likelihood ratio test. All analyses were performed using the R software (version 4.2.3). A *p*-value of <0.05 on a two-sided test was considered statistically significant.

## 3. Results

### 3.1. Participants Characteristics

Participants’ characteristics by total flavonoid intake levels are presented in Table 1. Of the 2581 participants, 1212 (46.96%) are male, with a mean (SD) age of 47.92 ± 0.92 years old. Participants with a higher intake of flavonoids are more likely to be female, non-smokers, non-drinkers, engage in regular physical activity, and have a higher education level, higher family income, higher HEI-2015 score, and lower prevalence of CVD. In addition, Table S1 shows the characteristics of MASLD status. Of the 2581 participants, 555 (21.50%) are diagnosed with MASLD. Participants with MASLD are more likely to be older, male, former smokers, non-drinkers, diagnosed with CVD, have a higher metabolic syndrome score, engage in irregular physical activity, and experience poorer sleep quality.

### 3.2. Measurement of Flavonoid Intake and Their Correlation

**Figure 2:**
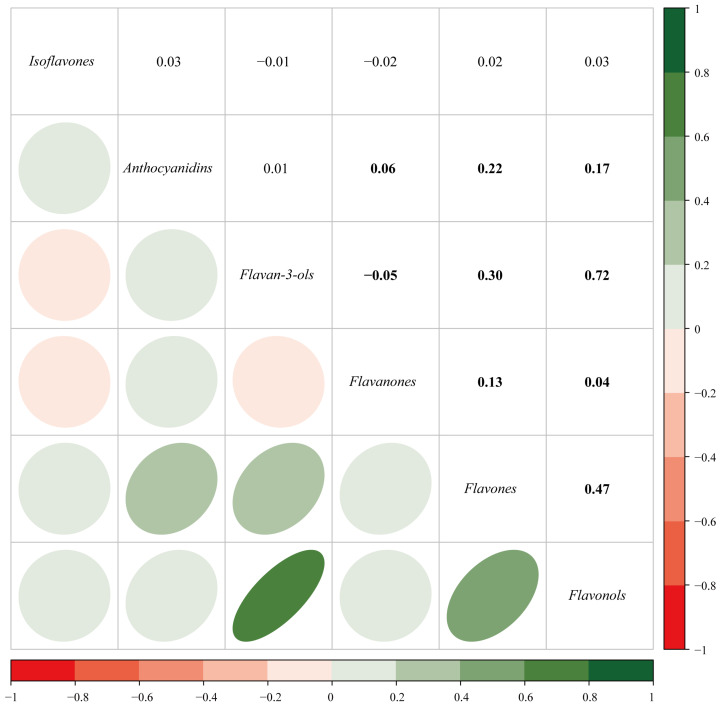
Pairwise Pearson correlations among six subclasses of flavonoids. The bolded numbers indicate statistically significant.

**Figure 3:**
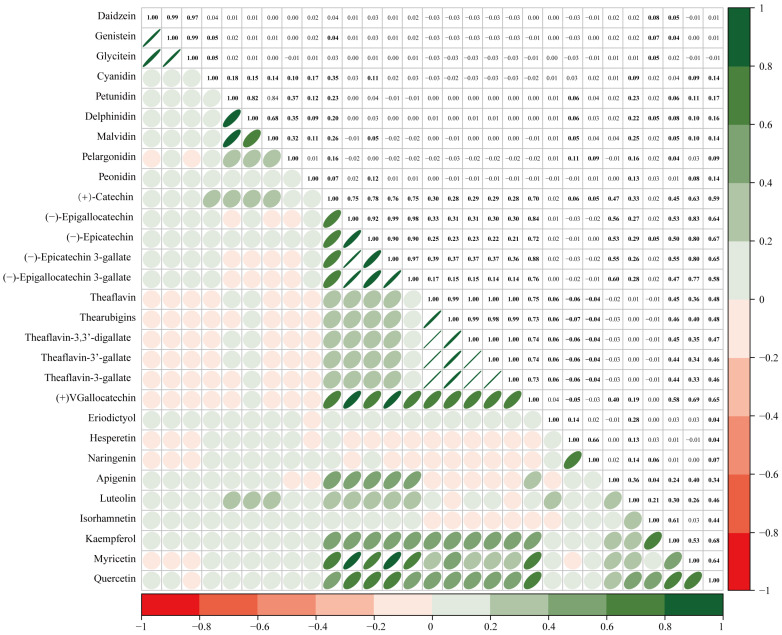
Pairwise Pearson correlations among 29 single components in participants. The bolded numbers indicate statistically significant.

All six subclasses of flavonoids and their individual components were analysed in the present study. The mean concentrations and distributions of these flavonoids (both raw and energy-adjusted values) are listed in Table S2. Statistically significant correlations are found between the concentrations of some of the six subclasses (Figure 2). Strong associations are observed between flavan-3-ols and flavonols (r = 0.72), flavones and flavonols (r = 0.47), as well as flavan-3-ols and flavones (r = 0.30). The other associations are relatively weak. Furthermore, slight to strong correlations are also found between the individual components (Figure 3, r = −0.04 to −0.07 and 0.04 to 1.00, *p* < 0.05).

### 3.3. Association Between Total Dietary Flavonoid Intake and MASLD

Table 2 illustrates the association between total dietary flavonoid intake and MASLD. Compared with the lowest tertile group, participants in the highest tertile of total dietary flavonoid intake have a lower risk of MASLD after multivariable adjustments (OR: 0.71, 95% CI: 0.51–0.97).

### 3.4. Interaction Between Total Flavonoid Intake and Selected Covariates

As shown in Table S3, the stratified analysis demonstrates that there are no statistically significant interactions between total flavonoid intake and most selected covariates (age, sex, smoking status, alcohol drinking status, metabolic syndrome score, education level, physical activity, family income-to-poverty ratio, and sleep quality) on the risk of MASLD (*p-*_interaction_ > 0.05). However, we observe significant multiplicative modification by CVDs for total flavonoid intake, indicating that participants without CVDs seem to benefit more from sufficient flavonoid intake.

### 3.5. Combined Effects of Dietary Flavonoid Intake on MASLD

**Figure 4:**
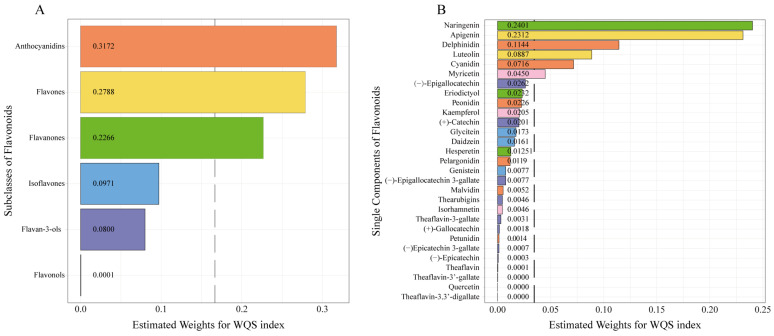
(**A**) WQS model regression index weights of six subclasses of flavonoids for MASLD. (**B**) WQS model regression index weights of 29 single components of flavonoids for MASLD. The analysis was adjusted for age, sex, ethnicity, smoking status, alcohol drinking status, metabolic syndrome score, cardiovascular disease, sleep quality, education level, family income-to-poverty ratio, regular physical activity, and the Healthy Eating Index 2015 score. Abbreviations: WQS, weighted quantile sum; MASLD, metabolic dysfunction associated with steatotic liver disease.

The WQS analysis was conducted to investigate the combined effects of six subclasses and 29 individual components of flavonoids on MASLD and to identify the predominant contributors. As shown in Tables S4 and S5, after fully adjusting, the WQS model demonstrates the mixed effects of six subclasses (OR: 0.63, 95% CI: 0.46–0.85) and 29 individual components (OR: 0.67, 95% CI: 0.49–0.92) are inversely associated with MASLD, which is consistent with the results presented in Table 2. Among the effects of the six subclasses on MASLD (Figure 4A), the most critical contributors are anthocyanidins (31.7%), flavones (27.9%), and flavanones (22.7%). As for the effects of 29 individual components of flavonoids on MASLD (Figure 4B), the most significant contributors are naringenin (24.0%), apigenin (23.1%), delphinidin (11.4%), luteolin (8.9%), cyanidin (7.2%), and myricetin (4.5%).

### 3.6. Individual Effects of Dietary Flavonoid Intake on MASLD

**Figure 5:**
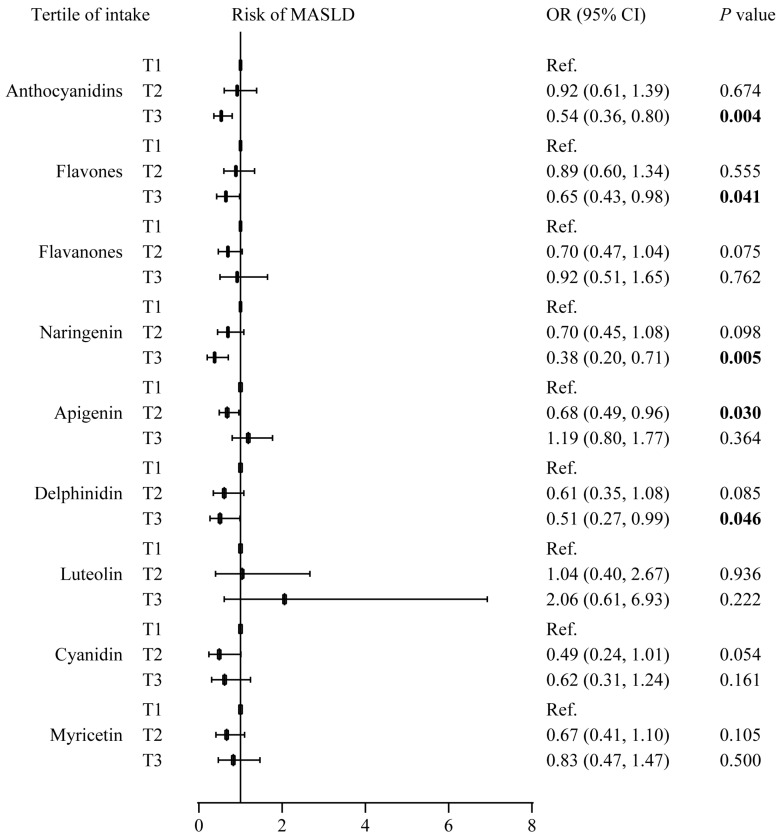
Analysis of predominant contributors in flavonoids and the risk of MASLD. All the analyses were adjusted for age, sex, ethnicity, smoking status, alcohol drinking status, metabolic syndrome score, cardiovascular disease, sleep quality, education level, family income-to-poverty ratio, regular physical activity, and the Healthy Eating Index 2015 score. The associations between anthocyanidins, flavones, flavanones, and the risk of MASLD were additionally adjusted for total flavonoid intake. The associations between anthocyanidins, flavones, flavanones, and the risk of MASLD were additionally adjusted for total flavonoid intake. The association between naringenin and the risk of MASLD was additionally adjusted for flavanones intake. The associations between apigenin, luteolin, and the risk of MASLD were additionally adjusted for flavone intake. The associations between delphinidin, cyaniding, and the risk of MASLD were additionally adjusted for anthocyanidin intake. The association between myricetin and the risk of MASLD was additionally adjusted for flavonol intake. Abbreviations: MASLD, metabolic dysfunction associated steatotic liver disease; OR, odds ratio; CI, confidence Interval.

Figure 5 illustrates the individual effects of predominant contributors in flavonoids on the risk of MASLD after multivariable adjustments. Compared with the lowest tertile of anthocyanidins, flavones, naringenin, apigenin, and delphinidin intake, participants with higher intakes have a lower risk of MASLD (OR = 0.38 to 0.68).

## 4. Discussion

To the best of our knowledge, this study is the first to underscore the protective effects of multiple and individual dietary flavonoids against MASLD in American adults. Our findings revealed that a higher total flavonoid intake was associated with a reduced risk of MASLD. Anthocyanins accounted for 31.7% of the contribution of flavonoid intake in reducing the risk of MASLD. Naringenin, apigenin, and delphinidin ranked highest among 29 individual components, with 24.0%, 23,1%, and 11.4% contribution of total flavonoid intake, respectively. Individual effects of anthocyanidins, flavones, naringenin, apigenin, and delphinidin against MASLD were also observed. We recommend prioritizing the flavonoids with the most significant contribution to MASLD prevention in our diet.

Pathologically, several biological mechanisms contribute to the development of MASLD, including autophagy [29], ferroptosis [30], insulin resistance [31], inflammation [32], and oxidative stress [33]. Among these, oxidative stress has been identified as the most important and universal pathway. For example, Klisic et al. found that pro-oxidants, such as advanced oxidation protein products, total oxidant status, and oxidative stress index, significantly increased in participants with NAFLD [34]. Meanwhile, previous studies revealed that the severity of NAFLD is correlated with an increase in oxidative stress [35], and with a significant upregulation of advanced glycation end products, which are products of oxidative damage, in cases of moderate steatosis compared with minimal steatosis [36]. Furthermore, both in vitro and in vivo experimental studies have demonstrated that the elevation of the hepatic regulator of G protein signalling six could promote ROS production to aggravate oxidative stress, which in turn drives the progression of MASLD [37]. Given the pivotal roles of oxidative stress in MASLD, it is reasonable to postulate that targeting oxidative stress reduction may serve as a potential therapeutic strategy to prevent the initiation and progression of MASLD.

Nutritional modulation has long been proposed as a promising approach to ameliorate NAFLD, due to its strengths of being cost-effective, safe, and easily conducted, as well as highly enriched phytochemicals and antioxidative nutrients in certain foods. For instance, it was reported that high adherence to a Mediterranean diet could inhibit oxidative stress-related biomarkers [38], and was associated with decreased BMI, body weight, WC, DBP, and SBP in participants with NAFLD [39]. However, whether these strategies could be applied in MASLD prevention remains unknown. Flavonoids are a class of natural compounds that consists of six subclasses and 29 individual components, which are enriched in dietary sources such as vegetables and fruits. Many studies have demonstrated their favourable roles in disease prevention due to their antioxidative effects. For example, in the offspring of mice exposed to bisphenol A, flavonoids could reduce oxidative stress to improve sperm damage [40]. In endothelial cells, herbal medicines-derived flavonoids could be used as anti-atherogenic agents by reducing oxidative stress [41]. Meanwhile, several experimental studies demonstrated the protective effects of flavonoids on liver diseases, such as liver injury [42], NAFLD [43], liver fibrosis [44], and liver cancer [45]. Epidemiological studies revealed that a higher flavonoid intake is associated with a low initiation and progression risk of NAFLD [15,46]. Moreover, a systematic review conducted by Naselli et al. provided a comprehensive understanding of the antioxidant mechanisms of flavonoids [47]. Since experimental evidence has highlighted the protective, antioxidant, and anti-inflammatory properties of flavonoids, the study suggested that flavonoids had the potential to be part of broader nutritional interventions for metabolic health. Therefore, we further explored the effects of flavonoids on MASLD. Our results for the first time illustrated that a higher flavonoid intake is associated with a lower risk of MASLD, indicating the protective roles of flavonoids in MASLD.

Although flavonoids exhibit protective effects on MASLD, do all subclasses and individual components contribute equally? In NAFLD, various studies consistently illustrated the protective roles of anthocyanidins and isoflavones, while the effect of flavones remains controversial [46,48,49]. In addition, regarding anthocyanidins, protective effects against NAFLD were only found in cyanidin and delphinidin [17]. Given the fact that different foods contain different levels of subclasses of flavonoids, and the content of individual components in the foods also varies, it is necessary to explore which one contributes the most. This will aid in developing precise nutritional strategies for managing MASLD. In our study, WQS results revealed that the protective effects of anthocyanidins, flavones, naringenin, apigenin, and delphinidin rank highest among all flavonoids. Thus, a higher dietary intake of foods enriched in these subclasses or individual components may reduce the risk of MASLD. The foods richest in flavonoids with the strongest effects on MASLD are presented in Table S6.

This study has several notable strengths. First, the data were derived from a nationally representative sample of the U.S., which broadens the applicability of the results. Moreover, NHANES implemented stringent quality control measures during the data collection process to uphold its validity. Third, the multivariable models considered a wide range of possible intervening variables, including data related to sociodemographic characteristics, lifestyle habits, and medical conditions. Fourth, the VCTE examination has shown significant advantages in large-scale population hepatic steatosis screening due to its non-invasive, real-time, high sensitivity, specificity, and low-cost characteristics. Finally, compared with previous studies on NAFLD [15,46,48], our study is the first to analyse the contributions of six subclasses and 29 components of flavonoids to MASLD risk. These findings are crucial for the precise management of MASLD using nutritional strategies.

Nonetheless, three limitations should be acknowledged. First, since this is an observational study, we cannot infer the cause-and-effect relationships. Second, further studies, especially experimental investigations, are needed to confirm the protective effects of flavonoids on the occurrence and development of MASLD. Third, a recall basis existed because all the dietary information was collected from participants by self-reporting.

## 5. Conclusions

In summary, we found that higher dietary flavonoid intake is associated with a reduced risk of MASLD. Anthocyanidins and naringenin exhibited the most significant protective effects on MASLD among six subclasses and 29 components, respectively. Furthermore, flavones, flavanones, apigenin, delphinidin, and myricetin were also recommended as a priority, due to their large contribution to MASLD protection. Further studies are warranted to validate our findings and explore the underlying mechanisms.

## Figures and Tables

**Table 1:** Characteristics of participants by tertile groups of total flavonoid intake.

Characteristics	Total (*n* = 2581)	T1 (*n* = 861)	T2 (*n* = 860)	T3 (*n* = 860)	*p*-Value
Age (year)	47.92 (0.92)	45.65 (1.32)	49.71 (1.18)	48.45 (1.04)	0.056
Sex, *n* (%)					<0.001
Male	1212 (46.96)	480 (54.57)	346 (38.86)	386 (46.38)	
Female	1369 (53.04)	381 (45.43)	514 (61.14)	474 (53.62)	
Ethnicity, *n* (%)					0.008
Mexican American	308 (11.93)	114 (9.94)	128 (10.51)	66 (5.48)	
Non-Hispanic White	922 (35.72)	332 (62.58)	273 (59.41)	317 (65.60)	
Non-Hispanic Black	590 (22.86)	229 (13.86)	207 (11.38)	154 (7.16)	
Other race	761 (29.48)	186 (13.62)	252 (18.71)	323 (21.76)	
Smoking status, *n* (%)					0.004
Never	1587 (61.49)	453 (54.02)	536 (66.57)	598 (70.52)	
Ever	624 (24.18)	225 (28.28)	226 (23.00)	173 (19.27)	
Current	370 (14.34)	183 (17.70)	98 (10.43)	89 (10.21)	
Alcohol drinking status, *n* (%)					0.010
Non-drinker	298 (11.55)	53 (5.35)	117 (10.81)	128 (11.82)	
Low to moderate drinker	2283 (88.45)	808 (94.65)	743 (89.19)	732 (88.18)	
Education level, *n* (%)					0.009
Less than high school	425 (16.47)	154 (10.81)	168 (10.78)	103 (6.96)	
High school or equivalent	584 (22.63)	236 (34.18)	194 (24.27)	154 (23.86)	
College or above	1572 (60.91)	471 (55.02)	498 (64.95)	603 (69.18)	
Diagnosed with cardiovascular disease, *n* (%)					0.022
Yes	286 (11.08)	100 (7.13)	105 (9.92)	81 (6.03)	
No	2295 (88.92)	761 (92.87)	755 (90.08)	779 (93.97)	
Metabolic syndrome score	1.95 (0.07)	1.99 (0.11)	1.99 (0.07)	1.88 (0.10)	0.439
Regular physical activity, *n* (%)					0.016
Yes	1251 (48.47)	325 (48.09)	441 (59.66)	485 (59.12)	
No	1330 (51.53)	536 (51.91)	419 (40.34)	375 (40.88)	
Healthy Eating Index 2015 score	52.75 (0.79)	46.60 (0.64)	55.12 (1.05)	56.34 (0.99)	<0.001
Sleep quality, *n* (%)					0.819
Low	94 (3.64)	29 (3.09)	36 (4.11)	29 (3.38)	
Moderate	761 (29.48)	268 (29.25)	269 (30.24)	224 (27.28)	
High	1726 (66.87)	564 (67.66)	555 (65.65)	607 (69.34)	
Family income-to-poverty ratio, *n* (%)					0.004
<1.0	388 (15.03)	142 (13.23)	142 (11.32)	104 (8.88)	
1.0–3.0	1194 (46.26)	470 (43.10)	398 (36.24)	326 (31.62)	
>3.0	999 (38.71)	249 (43.67)	320 (52.44)	430 (59.50)	

**Table 2:** Association between total flavonoid intake and the risk of MASLD.

	OR (95% CI)	*p*-Value
Model 1		
T1	1.00 (Ref.)	
T2	0.96 (0.64, 1.44)	0.825
T3	0.67 (0.45, 1.00)	0.050
Model 2		
T1	1.00 (Ref.)	
T2	1.10 (0.76, 1.59)	0.598
T3	0.71 (0.51, 0.97)	0.036

Model 1 was adjusted for: age and sex. Model 2 was adjusted for: age, sex, ethnicity, smoking status, alcohol drinking status, metabolic syndrome score, cardiovascular disease, sleep quality, education level, family income-to-poverty ratio, regular physical activity, and the Healthy Eating Index 2015 score. Abbreviations: MASLD, metabolic dysfunction associated steatotic liver disease; OR, odds ratio; CI, confidence interval.

## Data Availability

All data are available on request.

## Supplementary Materials

The following supporting information can be downloaded at: https://www.mdpi.com/article/10.3390/nu17020205/s1, Table S1: Characteristics of participants (N = 2581); Table S2. Raw and energy-adjusted values of dietary flavonoid intake (mg/day); Table S3. Interaction between total flavonoid intake and selected covariates; Table S4. Association between WQS index of six subclasses and MASLD risk; Table S5. Association between WQS index of 29 individual components of flavonoids and MASLD risk; Table S6. The foods richest in predominant types of flavonoids for MASLD prevention.

## Abbreviations

BMI: body mass index; CAP, controlled attenuation parameter; CI, confidence interval; CVD, cardiovascular diseases; FBG, fasting blood-glucose; FNDDS, Food and Nutrient Database for Dietary Studies; HEI-2015, HBV, hepatitis B virus; HCV, hepatitis C virus; Healthy Eating Index 2015; MASLD, metabolic dysfunction associated with steatotic liver disease; MEC, Mobile Examination Center; MetALD, metabolic dysfunction–associated steatotic liver disease; NAFLD, nonalcoholic fatty liver disease; NHANES, National Health and Nutrition Examination Survey; OR, odds ratio; SD, standard deviation; SLD, steatotic liver diseases; WC, waist circumference; WQS, weighted quantile sum; VCTE, vibration-controlled transient elastography.
