# Supplementary material for: Associations of the Intake of Individual and Multiple Flavonoids with Metabolic Dysfunction Associated Steatotic Liver Disease in the United States
Source: Nutrients. 2025 Jan 7;17(2):205. doi: 10.3390/nu17020205 (PMC11768006; doi:10.3390/nu17020205)
Supplement: Supplementary file 1 [file nutrients-17-00205-s001.zip › nutrients-3389900-supplementary.pdf]

Table S1. Characteristics of participants (N=2581).

Characteristcs	Total (n=2581)	Without MASLD (n=2026)	With MASLD (n=555)	*P* value
**Age (year)**	47.92 (0.92)	46.34 (1.03)	54.90 (0.90)	< 0.001
**Sex, n (%)**				< 0.001
Male	1212 (46.96)	897 (44.19)	315 (57.79)	
Female	1369 (53.04)	1129 (55.81)	240 (42.21)	
**Ethnicity, n (%)**				0.477
Mexican American	308 (11.93)	235 (8.77)	73 (7.51)	
Non-Hispanic White	922 (35.72)	703 (62.20)	219 (64.63)	
Non-Hispanic Black	590 (22.86)	492 (11.13)	98 (8.78)	
Other race	761 (29.48)	596 (17.89)	165 (19.09)	
**Smoking status, n (%)**				0.018
Never	1587 (61.49)	1270 (64.78)	317 (59.67)	
Ever	624 (24.18)	443 (21.50)	181 (31.88)	
Current	370 (14.34)	313 (13.72)	57 (8.44)	
**Alcohol drinking status, n (%)**				0.002
Nondrinker	298(11.55)	213(7.53)	85(17.49)	
Low to moderate drinker	2283(88.45)	1813(92.47)	470(82.51)	
**Education level, n (%)**				0.622
Less than high school	425 (16.47)	336 (9.39)	89 (9.64)	
High school or equivalent	584 (22.63)	446 (26.79)	138 (30.04)	
College or above	1572 (60.91)	1244 (63.82)	328 (60.32)	
**Diagnosed with cardiovascular disease , n (%)**				0.006
Yes	286 (11.08)	203 (6.80)	83 (11.25)	
No	2295 (88.92)	1823 (93.20)	472 (88.75)	
**Metabolic syndrome score**	1.95 (0.07)	1.67 (0.06)	3.18 (0.11)	< 0.001
**Regular physical activity, n (%)**				< 0.001
Yes	1251 (48.47)	1040 (59.04)	211 (40.70)	
No	1330 (51.53)	986 (40.96)	344 (59.30)	
**Healthy Eating Index 2015 score**	52.75 (0.79)	53.20 (0.88)	50.75 (1.07)	0.065
**Sleep quality, n (%)**				0.002
Low	94 (3.64)	61 (3.30)	33 (4.47)	
Moderate	761 (29.48)	581 (27.02)	180 (36.97)	
High	1726 (66.87)	1384 (69.67)	342 (58.56)	
**Family income-to-poverty ratio, n (%)**				0.550
<1.0	388 (15.03)	307 (10.85)	81 (12.11)	
1.0-3.0	1194 (46.26)	936 (36.41)	258 (38.88)	
>3.0	999 (38.71)	783 (52.74)	216 (49.01)	

Abbreviations: MASLD, metabolic dysfunction associated steatotic liver disease.

Table S2. Raw and energy-adjusted values of dietary flavonoids intake (mg/day).

	**Flavonoids intake without energy-adjusted**		**Flavonoids intake with energy-adjusted**	
	**Without MASLD**	**With MASLD**	**Without MASLD**	**With MASLD**
Total Flavonoids intake	215.19 (15.33)	231.00 (32.83)	213.72 (14.91)	225.73 (33.66)
Total Isoflavones Intake	3.31 (0.58)	1.38 (0.59)	3.30 (0.58)	1.32 (0.59)
Total Anthocyanidins Intake	16.70 (2.10)	9.15 (1.22)	16.56 (2.06)	8.65 (1.26)
Total Flavan-3-ols intake	165.23 (13.91)	192.05 (31.98)	164.15 (13.65)	188.16 (32.58)
Total Flavanones Intake	10.74 (1.00)	9.76 (0.89)	10.65 (0.98)	9.44 (0.92)
Total Flavones Intake	0.96 (0.06)	0.70 (0.06)	0.96 (0.05)	0.68 (0.06)
Total Flavonols Intake	18.23 (0.64)	17.97 (1.01)	18.10 (0.58)	17.48 (1.05)
Cyanidin	2.67 (0.49)	2.43 (0.73)	2.65 (0.49)	2.33 (0.73)
Delphinidin	2.22 (0.35)	1.09 (0.19)	2.21 (0.34)	1.05 (0.19)
Malvidin	5.36 (0.76)	2.42 (0.37)	5.33 (0.75)	2.32 (0.38)
Pelargonidin	1.58 (0.20)	1.09 (0.18)	1.56 (0.20)	1.03 (0.19)
Peonidin	3.32 (0.56)	1.32 (0.30)	3.27 (0.54)	1.16 (0.30)
Petunidin	1.56 (0.25)	0.79 (0.15)	1.55 (0.25)	0.76 (0.15)
(-)-Epicatechin	18.28 (1.83)	18.51 (2.67)	10.21 (0.65)	10.00 (0.62)
(-)-Epicatechin 3-gallate	10.29 (0.67)	10.27 (0.61)	11.28 (1.09)	11.14 (2.06)
(-)-Epigallocatechin	11.33 (1.10)	11.32 (2.04)	18.20 (1.81)	18.21 (2.69)
(-)-Epigallocatechin 3-gallate	32.39 (3.91)	27.87 (5.04)	32.29 (3.90)	27.48 (5.06)
(+)-Catechin	7.67 (0.47)	6.81 (0.48)	7.61 (0.45)	6.60 (0.52)
(+)-Gallocatechin	1.58 (0.15)	1.81 (0.37)	1.57 (0.14)	1.78 (0.38)
Theaflavin	1.40 (0.19)	1.93 (0.41)	1.39 (0.19)	1.89 (0.42)
Theaflavin-3,3'-digallate	1.53 (0.21)	2.12 (0.46)	1.51 (0.21)	2.07 (0.46)
Theaflavin-3'-gallate	1.30 (0.18)	1.82 (0.39)	1.29 (0.18)	1.78 (0.40)
Theaflavin-3-gallate	1.08 (0.15)	1.50 (0.33)	1.07 (0.15)	1.47 (0.33)
Thearubigins	78.38 (9.76)	108.07 (20.93)	77.73 (9.65)	105.74 (21.37)
Eriodictyol	0.13 (0.02)	0.12 (0.05)	0.13 (0.02)	0.11 (0.05)
Hesperetin	7.29 (0.61)	6.73 (0.54)	7.21 (0.59)	6.45 (0.57)
Naringenin	3.32 (0.50)	2.91 (0.48)	3.31 (0.50)	2.88 (0.49)
Apigenin	0.18 (0.02)	0.12 (0.01)	0.18 (0.02)	0.11 (0.01)
Luteolin	0.79 (0.04)	0.58 (0.06)	0.78 (0.04)	0.56 (0.06)
Isorhamnetin	0.96 (0.05)	0.81 (0.07)	0.95 (0.05)	0.78 (0.07)
Kaempferol	4.72 (0.22)	4.34 (0.43)	4.69 (0.21)	4.22 (0.46)
Myricetin	1.64 (0.08)	1.87 (0.17)	1.63 (0.08)	1.83 (0.17)
Quercetin	10.92 (0.38)	10.95 (0.59)	10.83 (0.34)	10.65 (0.61)
Daidzein	1.23 (0.21)	0.49 (0.19)	1.22 (0.21)	0.46 (0.19)
Genistein	1.81 (0.32)	0.78 (0.35)	1.80 (0.32)	0.75 (0.35)
Glycitein	0.28 (0.05)	0.11 (0.05)	0.28 (0.05)	0.11 (0.05)

Abbreviations: MASLD, metabolic dysfunction associated steatotic liver disease.

Table S3. Interaction between total flavonoid intake and selected covariates

	OR (95% CI)			*P*-interaction
	**T1**	**T2**	**T3**	
**Age**				0.379
<60	1.00 (Ref.)	1.37 (0.96, 1.96)	0.65 (0.41, 1.05)	
≥60	1.00 (Ref.)	0.85 (0.37, 1.97)	0.78 (0.39, 1.59)	
**Sex**				0.623
Male	1.00 (Ref.)	1.18 (0.81, 1.71)	0.66 (0.48, 0.92)	
Female	1.00 (Ref.)	1.03 (0.54, 1.98)	0.76 (0.39, 1.49)	
**Smoking status**				0.760
Never	1.00 (Ref.)	0.96 (0.60, 1.52)	0.70 (0.43, 1.13)	
Ever	1.00 (Ref.)	1.20 (0.61, 2.35)	0.61 (0.28, 1.33)	
Current	1.00 (Ref.)	1.61 (0.55, 4.68)	0.77 (0.27, 2.24)	
**Alcohol drinking status**				0.142
Nondrinker	1.00 (Ref.)	0.52 (0.18, 1.52)	0.32 (0.12, 0.80)	
Low to moderate drinker	1.00 (Ref.)	1.25 (0.88, 1.77)	0.79 (0.59, 1.04)	
**Metabolic syndrome score**				0.618
T1	1.00 (Ref.)	1.37 (0.57, 3.30)	1.07 (0.33, 3.47)	
T2	1.00 (Ref.)	1.27 (0.80, 2.04)	0.91 (0.55, 1.51)	
T3	1.00 (Ref.)	1.10 (0.63, 1.91)	0.50 (0.24, 1.06)	
**Education level**				0.391
Less than high school	1.00 (Ref.)	2.03 (1.15, 3.57)	0.85 (0.25, 2.86)	
High school or equivalent	1.00 (Ref.)	1.19 (0.64, 2.21)	1.19 (0.52, 2.69)	
College or above	1.00 (Ref.)	0.98 (0.57, 1.71)	0.57 (0.34, 0.95)	
**Regular physical activity**				0.548
Yes	1.00 (Ref.)	0.79 (0.43, 1.45)	0.51 (0.28, 0.91)	
No	1.00 (Ref.)	1.34 (0.77, 2.34)	0.89 (0.59, 1.35)	
**Diagnosed with cardiovascular disease**				0.009
Yes	1.00 (Ref.)	1.59 (0.61, 4.15)	3.59 (0.98, 13.20)	
No	1.00 (Ref.)	1.05 (0.68, 1.62)	0.60 (0.42, 0.85)	
**Family income-to-poverty ratio**				0.923
<1.0	1.00 (Ref.)	0.91 (0.38, 2.21)	0.71 (0.24, 2.14)	
1.0-3.0	1.00 (Ref.)	0.90 (0.63, 1.30)	0.57 (0.38, 0.85)	
>3.0	1.00 (Ref.)	1.14 (0.56, 2.31)	0.83 (0.53, 1.31)	
**Healthy Eating Index 2015 score**				0.105
T1	1.00 (Ref.)	0.51 (0.26, 1.00)	0.75 (0.47, 1.21)	
T2	1.00 (Ref.)	1.67 (0.66, 4.21)	0.77 (0.33, 1.81)	
T3	1.00 (Ref.)	1.43 (0.73, 2.77)	0.74 (0.39, 1.40)	
**Sleep quality**				0.542
Low	1.00 (Ref.)	0.02 (0.00, 1.45)	0.05 (0.00, 0.77)	
Moderate	1.00 (Ref.)	1.53 (0.76, 3.09)	1.12 (0.68, 1.84)	
High	1.00 (Ref.)	0.99 (0.61, 1.60)	0.66 (0.36, 1.23)	

Abbreviations: OR, odds ratio; CI, confidence interval.

Table S4. Association between WQS index of six subclasses and MASLD risk

**Outcomes**	**OR (95% CI)**	***P* value**
Model 1	0.63 (0.50, 0.79)	0.007
Model 2	0.63 (0.46, 0.85)	0.003

Model 1 was adjusted for: age, sex.

Model 2 was adjusted for: age, sex, ethnicity, smoking status, alcohol drinking status, metabolic syndrome score, cardiovascular disease, sleep quality, education level, family income-to-poverty ratio, regular physical activity, Healthy Eating Index 2015 score.

Abbreviations: MASLD, metabolic dysfunction associated steatotic liver disease; OR: odds ratio; CI: confidence interval; WQS, weighted quantile sum.

Table S5. Association between WQS index of 29 individual components of flavonoids and MASLD risk

**Outcomes**	**OR (95% CI)**	***P* value**
Model 1	0.66 (0.52, 0.85)	0.011
Model 2	0.67 (0.49, 0.92)	0.015

Model 1 was adjusted for: age, sex.

Model 2 was adjusted for: age, sex, ethnicity, smoking status, alcohol drinking status, metabolic syndrome score, cardiovascular disease, sleep quality, education level, family income-to-poverty ratio, regular physical activity, Healthy Eating Index 2015 score.

Abbreviations: MASLD, metabolic dysfunction associated steatotic liver disease; OR: odds ratio; CI: confidence interval; WQS, weighted quantile sum.

Table S6. The foods richest in predominant types of flavonoids for MASLD prevention.

Predominant flavonoids with the most significant contribution to MASLD prevention	Foods that are abundant in specific flavonoids
Anthocyanidins	Acai, Arctic bramble berry, Bilberry, Blackberry, Black currant, Chokeberry, Currant, Elderberry
Flavones	Acai, Arctic bramble berry, Bilberry, Blackberry, Black currant, Chokeberry, Currant, Elderberry
Flavanones	Grapefruit, Kumquat, Lemon, Lime, Oregano, Peppermint, Pummelo, Sour orange, Tangelo, Tangor
Naringenin	Grapefruit, Kumquat, Oregano, pummelo, Rosemary, Sour orange, tangelo, Yuzu
Apigenin	Artichoke, Celery, Celery seed, Juniper berry, Kumquat, Oregano, Parsley, Sorghum, Thyme
Delphinidin	Bilberry, Blackberry, Currant, Eggplant, Grape, Maqui, Service berry, Black currant
Myricetin	Black currant, Bog whortleberry, Carob fiber, Carob kibble, Cranberry, Fennel, Goji berry, Parsley

According to the data published by the United States Department of Agriculture, foods with high number of flavonoids per 100g are recommended. More information on the flavonoids composition of foods can be obtained from website: https://fdc.nal.usda.gov.
